# A Web Screening on Educational Initiatives to Increase Citizens’ Literacy on Genomics and Genetics

**DOI:** 10.3389/fgene.2021.637438

**Published:** 2021-07-07

**Authors:** Michele Sassano, Giovanna Elisa Calabrò, Stefania Boccia

**Affiliations:** ^1^Section of Hygiene, Department of Life Sciences and Public Health, Università Cattolica del Sacro Cuore, Rome, Italy; ^2^Department of Woman and Child Health and Public Health – Public Health Area, Fondazione Policlinico Universitario A. Gemelli IRCCS, Rome, Italy

**Keywords:** citizens, literacy, omics sciences, personalized medicine, initiatives

## Abstract

**Introduction:**

Population awareness and empowerment in omics sciences represent a fundamental driver to increase the adoption of evidence-based approaches in personalized medicine. In this context, a pivotal role is played by citizens’ literacy, and educational initiatives carried out in this context are key assets to drive future effective interventions. With the present study, we summarized the educational initiatives conducted worldwide aimed at increasing citizens’ literacy in omics sciences.

**Materials and Methods:**

We conducted a web search of the educational initiatives aimed at improving citizens’ literacy in omics sciences undertaken worldwide, by using three search engines (Google, Bing, and Yahoo Search), in English and in Italian languages.

**Results:**

We identified five initiatives in Europe, 22 in non-European countries, and 13 in Italy. Overall, the majority (69%) were web-based initiatives, while 31% required in-person attendance. The online initiatives included web pages for reading, online lessons/courses, web portals, videos/short movies, animations, and apps for mobile devices. The residential initiatives, on the other hand, included exhibitions, seminars, courses, symposia, information stands in public places, guided visits to research laboratories, and interactive laboratories. All the initiatives were highly heterogeneous in terms of methodologies and the topics addressed.

**Discussion and Conclusion:**

Overall, we identified a variety of initiatives aimed at improving citizens’ literacy in omics sciences, with the largest majority carried out in the United States and being web-based. Our results showed heterogeneity among the initiatives as to the dealt topics and the adopted methods. Further research is needed, however, to quantitatively assess the effectiveness of educational initiatives to improve citizens’ literacy in omics sciences.

## Introduction

Advancements in the omics field promise a new era of personalized medicine (PM) in healthcare. A major promise of the “omics” research is that of delivering new information that can transform healthcare through earlier diagnosis, more effective prevention programs, and a higher precision in the treatment of disease (Boccia, 2014). Even though the integration of PM into practice is yet to happen in many health systems and countries worldwide, the exponential growth of knowledge in this field, the increasing costs of new technologies, and, sometimes, the lack of regulation make public health and health systems face a number of challenges (Ricciardi and Boccia, 2017). Among them, health systems should be prepared to face such a profound change in healthcare in order to allow for a better alignment of current research and clinical practice and to allow equitable access to new practices to all citizens and patients. In addition, the adoption of omics technologies and practices will require citizens to be appropriately aware of their benefits, risks, and real utility. This might be achieved through the improvement of literacy of healthcare professionals and citizens (Etchegary and Wilson, 2013; Calabrò et al., 2020). Increasing citizens’ literacy requires not only specific initiatives aimed at the appropriate and conscious utilization of the new “omics” technologies but also correct information of users, for example, on the direct-to-consumer genetic tests (DTC-GTs) (Pearce et al., 2019; Hoxhaj et al., 2020; Pastorino et al., 2021). Educational initiatives are therefore needed to allow citizens to acquire correct and reliable information on both the benefits and possible risks of PM in order to make appropriate health decisions supported by healthcare professionals (Ricciardi and Boccia, 2017) and to become active players in the decision-making process (Etchegary and Wilson, 2013), as already highlighted in the *Vision Paper on Personalised Medicine Research and Implementation by 2030* from the International Consortium for Personalised Medicine (ICPerMed) (International Consortium for Personalised Medicine, 2019).

To date, the landscape of existing citizens’ literacy initiatives on omics sciences across the world is fragmented and sparse, even though some efforts were put in place for their identification (Genomic Literacy Education and Engagement (GLEE) initiative, 2017). The current knowledge of such initiatives is urgently needed across Europe, however, in order to design future educational initiatives that build up on a common knowledge base. In this context, national authorities in Europe are paying great attention to citizens’ literacy in omics sciences. As an example, Genomics England has been carrying out several public engagement activities in the United Kingdom over recent years (Samuel and Farsides, 2018). As for Italy, this is witnessed by the National Plan for Innovation of the Health System based on omics sciences, which addresses literacy of all stakeholders as a prerequisite for the correct implementation of omics sciences into practice (Boccia et al., 2017). To this aim, we attempted to summarize all the educational initiatives aimed at improving citizens’ literacy in the field of omics sciences in the context of a project funded by the National Center for Disease Prevention and Control (CCM) of the Italian Ministry of Health through a web screening of ongoing and past initiatives worldwide, with a particular focus on Italy and English-speaking countries.

## Materials and Methods

### Search Strategy

We conducted a web search of online and in-person educational initiatives carried out in European and non-European countries aimed at educating citizens in the field of omics sciences without limit of the age of the target population. An additional focus was dedicated to Italian initiatives.

The search was conducted using the three most used web search engines worldwide: Google, Bing, and Yahoo Search (Statista, 2021). The search was limited to articles published in English and Italian languages and was performed in June 2020.

We used the following terms for the web search in Google using its “advanced search” application^1^ : (genetics OR genomics OR omics sciences) AND education AND initiatives AND citizens. The search was repeated with the same terms in the Italian language, as follows: (genetica OR genomica OR scienze omiche) AND formazione AND iniziative AND cittadini. This search strategy was also used as the template for the search in other search engines.

After the launch of the search through the string, we filtered the results according to the categories “all” and “news” in order to find textual records relevant to our research aim and eligibility criteria, and no limits according to file type or date of publication were applied.

Two researchers (GC and MS) independently screened the identified records by title, abstract, and summary, whenever available, in order to identify the eligible initiatives. A database of relevant records from the screening stage was created using an Excel spreadsheet, and full texts or full web pages of these records were further assessed against our research aim and our eligibility criteria by two researchers (GC and MS) independently. Any discrepancy on the inclusion of the identified records was solved by discussion or by the involvement of a third researcher (SB).

Starting from the relevant pages identified, we performed a secondary search for other relevant initiatives that were suggested or mentioned on the web page using web links and articles retrieved at each web page. In addition, we manually searched the list of references of each relevant document and web page, if available.

### Eligibility Criteria

Eligible initiatives were those dealing with omics sciences and addressing citizens and those reporting the title and a minimum set of information including the target population, dealt topics, and aim. Initiatives aimed at students and teachers (up to high schools) were also included, while structured courses or degree courses promoted by universities were excluded.

### Data Extraction and Synthesis of Results

For each eligible initiative, two researchers (GC and MS) independently extracted the following information: name of initiative/project, country (and city for Italian initiatives requiring in-person attendance), period or year, organizer/promoter of the initiative, topic, type of initiative, target population, and type of attendance (in-person or digital). Any discrepancy in data extraction was solved by discussion, or with the involvement of a third researcher (SB) whenever agreement between the first two researchers (GC and MS) was not achieved through discussion.

We summarized the results using a narrative descriptive synthesis (Ryan, 2013), focusing on similarities and differences regarding the following extracted characteristics of the identified initiatives: topic, target population, and type of required attendance. These results were grouped and synthesized according to three categories: European initiatives excluding Italy, non-European initiatives, and Italian initiatives.

Preliminary findings were previously reported in brief elsewhere (Sassano et al., 2020). Here, we summarize the final results of our study.

## Results

**FIGURE 1:**
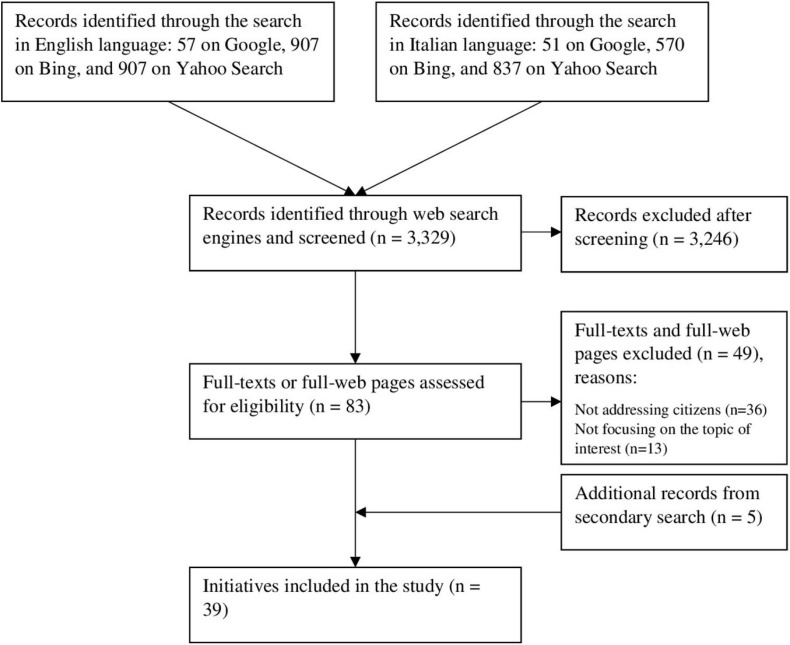
Flowchart of the selection process.

The search in English language produced 1,871 results (57 on Google, 907 on Bing, and 907 on Yahoo Search), while 1,458 results were yielded through the search in Italian language (51 on Google, 570 on Bing, and 837 on Yahoo Search). Details of the selection process are reported in the flowchart in Figure 1. After initial screening, 83 records were further assessed through examination of full texts or full web pages. Lastly, following in-depth examination of the identified records, we included 34 records, with five more identified through secondary search, thus leading to a total of 39 included initiatives: five conducted in Europe excluding Italy (yourgenome, 2017; GenoME, 2018; European Researchers’ Night, 2021; Navarrabiomed, 2021; Orphanet, 2021), 22 in non-European countries (HudsonAlpha, 2011, 2021; Genetic Science Learning Center of University of Utah Health Sciences, 2015, 2018; Yale University, 2016; 23andMe, 2021; Cold Spring Harbor Laboratory, 2021a,b; Columbia University Medical Center Division of Molecular Genetics, 2021; Department of Education of the American Museum of Natural History, 2021; DiseaseInfoSearch, 2021; Genes in Life, 2021; Genetic Literacy Project, 2021; Genome, 2021; GenomeQuébec, 2021; iBiology, 2021; MyGenome App, 2021; National Center for Advancing Translational Sciences, 2021; National Human Genome Research Institute, 2021a,b, e; Understanding Genetics, 2021), and 13 in Italy (European Researchers’ Night, 2021; Fondazione Telethon, 2021; Genetica biologia e salute, 2021; Istituto Italiano per la Medicina Genomica, 2021; Istituto Superiore di Sanità, 2021; Muse- Museo delle Scienze di Trento, 2021; Museo Tridentino di Scienze Naturali, 2021; Palazzo delle Esposizioni, 2021; Polo d’Innovazione di Genomica Genetica e Biologia, 2021; Portale Italiano delle Malattie Complesse, 2021; Scienze a Scuola, 2021; Università della Calabria, 2021; Zanichelli Aula di scienze, 2021; Supplementary Tables 1–5). One of the retrieved initiatives involved several countries, but since it is a web-based initiative, hence with no in-person events around the involved countries, it is reported only once in a single category, according to the country where it was originally founded (Orphanet, 2021).

The five initiatives conducted in European countries other than Italy addressed citizens/general population (yourgenome, 2017; GenoME, 2018; European Researchers’ Night, 2021; Navarrabiomed, 2021; Orphanet, 2021; Supplementary Tables 1, 2). Among the non-European ones, 15 initiatives addressed citizens/general population (HudsonAlpha, 2011, 2021; 23andMe, 2021; Cold Spring Harbor Laboratory, 2021a,b; Columbia University Medical Center Division of Molecular Genetics, 2021; DiseaseInfoSearch, 2021; Genes in Life, 2021; Genetic Literacy Project, 2021; iBiology, 2021; MyGenome App, 2021; National Center for Advancing Translational Sciences, 2021; National Human Genome Research Institute, 2021a,b; Understanding Genetics, 2021), six addressed students and/or teachers (Genetic Science Learning Center of University of Utah Health Sciences, 2015, 2018; Yale University, 2016; Department of Education of the American Museum of Natural History, 2021; GenomeQuébec, 2021; National Human Genome Research Institute, 2021e), and one involved both categories (Genome, 2021; Supplementary Tables 3, 4). Among the Italian initiatives, six addressed citizens/general population (European Researchers’ Night, 2021; Fondazione Telethon, 2021; Istituto Superiore di Sanità, 2021; Muse- Museo delle Scienze di Trento, 2021; Palazzo delle Esposizioni, 2021; Portale Italiano delle Malattie Complesse, 2021), six addressed students and/or citizens (Genetica biologia e salute, 2021; Istituto Italiano per la Medicina Genomica, 2021; Polo d’Innovazione di Genomica Genetica e Biologia, 2021; Scienze a Scuola, 2021; Università della Calabria, 2021; Zanichelli Aula di scienze, 2021), and one addressed both (Museo Tridentino di Scienze Naturali, 2021; Supplementary Tables 5, 6).

Overall, 31% (*n* = 12) of the retrieved initiatives required in-person attendance (Yale University, 2016; European Researchers’ Night, 2021; Genetica biologia e salute, 2021; Genome, 2021; Istituto Italiano per la Medicina Genomica, 2021; Muse- Museo delle Scienze di Trento, 2021; Museo Tridentino di Scienze Naturali, 2021; National Human Genome Research Institute, 2021e; Navarrabiomed, 2021; Palazzo delle Esposizioni, 2021; Polo d’Innovazione di Genomica Genetica e Biologia, 2021; Università della Calabria, 2021), including exhibitions, seminars, courses, symposia, information stands in public places, guided visits to research laboratories, and interactive laboratories, while 69% (*n* = 27) were web-based resources (HudsonAlpha, 2011, 2021; Genetic Science Learning Center of University of Utah Health Sciences, 2015, 2018; yourgenome, 2017; GenoME, 2018; 23andMe, 2021; Cold Spring Harbor Laboratory, 2021a,b; Columbia University Medical Center Division of Molecular Genetics, 2021; Department of Education of the American Museum of Natural History, 2021; DiseaseInfoSearch, 2021; Fondazione Telethon, 2021; Genes in Life, 2021; Genetic Literacy Project, 2021; GenomeQuébec, 2021; iBiology, 2021; Istituto Superiore di Sanità, 2021; MyGenome App, 2021; National Center for Advancing Translational Sciences, 2021; National Human Genome Research Institute, 2021a,b; Orphanet, 2021; Portale Italiano delle Malattie Complesse, 2021; Scienze a Scuola, 2021; Understanding Genetics, 2021; Zanichelli Aula di scienze, 2021). The latter were highly heterogeneous and included web pages for reading and consultation by the public, online lessons and courses, web portals aimed at giving information and advice, videos and short movies, animations, and apps for mobile devices. The identified initiatives focused mainly on genomics, in particular on the following topics: basic concepts of cellular biology and genetics, genetic risks of diseases, modern genome sequencing techniques, genetic tests, and the clustered regularly interspaced short palindromic repeats (CRISPR) technique, which is a gene editing tool (Supplementary Tables 1–5).

### European Initiatives

We identified five initiatives, of which two were performed in the United Kingdom (yourgenome, 2017; GenoME, 2018), two involved several European countries and cities (also non-European countries in one case) (European Researchers’ Night, 2021; Orphanet, 2021), and one was conducted in Spain (Navarrabiomed, 2021; Supplementary Table 1). Two initiatives required in-person attendance (European Researchers’ Night, 2021; Navarrabiomed, 2021), while three were web-based resources (yourgenome, 2017; GenoME, 2018; Orphanet, 2021), all involving the general population (Supplementary Table 1). One of the two in-person educational initiatives was promoted by the European Commission, with the 2018 and 2019 editions of “European Researchers’ Night” (European Researchers’ Night, 2021), which involved several cities across Europe with events focused on genetics, genomics, or omics sciences. In particular, four identified events were carried out in the United Kingdom, two in Ireland, two in Germany, one in Poland, and one in Spain. The project “European Researchers’ Night” allows the organization of scientific events every year, with the aim of making citizens more aware of science and of researchers’ daily activities and outputs (Supplementary Table 2). The second in-person event was organized by the Spanish company Navarrabiomed, which periodically organizes informative events open to citizens. An example is the event “¿Quieres visitar Navarrabiomed?” on November 6, 2019 (Navarrabiomed, 2021) that offered the general population the opportunity to visit a biomedical research center and understand the organization.

The three web-based resources identified are two websites and an application for tablets, both addressing the general population (yourgenome, 2017; GenoME, 2018; Orphanet, 2021; Supplementary Table 1). In detail, the interactive website yourgenome (2017) from the Public Engagement Team and scientists of the Wellcome Genome Campus, United Kingdom, is a resource for the general population to improve knowledge on genetics and genomics. The web platform hosts videos and interactive activities on a number of topics (e.g., DNA, genome sequencing, and DTC-GTs). On the other hand, the application GenoME (available only for Apple iPads) (GenoME, 2018) allows users to explore four Personal Genome Project United Kingdom ambassadors’ genetic codes and characteristics, for example, the ethnic origin, eye color, health, smoking habit, and age. Information are presented through animations and videos, and a musical interpretation of the genetic code can also be listened to by users. This application has the purpose of making citizens improve their knowledge about the human genome and understand how genetic variants could predict some phenotypic traits. Lastly, the Orphanet website is a web portal of rare diseases and orphan drugs, with the aim of spreading high-quality information among all the stakeholders. Orphanet was originally founded in France by the Institut National de la Santé et de la Recherche Médicale (INSERM) in 1997 and co-funded over the years by the European Commission, but gradually expanded to over 40 countries all over the world. Its website hosts an encyclopedia reporting information about rare diseases and the genes involved in their development, orphan drugs, patient associations, centers of excellence for the care of specific diseases, laboratories for the diagnosis of rare diseases, ongoing research projects, clinical trials, and biobanks.

### Non-European Initiatives

We identified 22 initiatives carried out in non-European countries (HudsonAlpha, 2011, 2021; Genetic Science Learning Center of University of Utah Health Sciences, 2015, 2018; Yale University, 2016; 23andMe, 2021; Cold Spring Harbor Laboratory, 2021a,b; Columbia University Medical Center Division of Molecular Genetics, 2021; Department of Education of the American Museum of Natural History, 2021; DiseaseInfoSearch, 2021; Genes in Life, 2021; Genetic Literacy Project, 2021; Genome, 2021; GenomeQuébec, 2021; iBiology, 2021; MyGenome App, 2021; National Center for Advancing Translational Sciences, 2021; National Human Genome Research Institute, 2021a,b, e; Understanding Genetics, 2021), and their characteristics are summarized in Supplementary Table 3. The vast majority were carried out in the United States (HudsonAlpha, 2011, 2021; Genetic Science Learning Center of University of Utah Health Sciences, 2015, 2018; Yale University, 2016; 23andMe, 2021; Cold Spring Harbor Laboratory, 2021a,b; Columbia University Medical Center Division of Molecular Genetics, 2021; Department of Education of the American Museum of Natural History, 2021; DiseaseInfoSearch, 2021; Genes in Life, 2021; Genetic Literacy Project, 2021; Genome, 2021; iBiology, 2021; MyGenome App, 2021; National Center for Advancing Translational Sciences, 2021; National Human Genome Research Institute, 2021a,b, e; Understanding Genetics, 2021), while one was in Canada (GenomeQuébec, 2021). Overall, three initiatives required in-person attendance (Yale University, 2016; Genome, 2021; National Human Genome Research Institute, 2021e), while 19 were web-based resources (HudsonAlpha, 2011, 2021; Genetic Science Learning Center of University of Utah Health Sciences, 2015, 2018; 23andMe, 2021; Cold Spring Harbor Laboratory, 2021a,b; Columbia University Medical Center Division of Molecular Genetics, 2021; Department of Education of the American Museum of Natural History, 2021; DiseaseInfoSearch, 2021; Genes in Life, 2021; Genetic Literacy Project, 2021; GenomeQuébec, 2021; iBiology, 2021; MyGenome App, 2021; National Center for Advancing Translational Sciences, 2021; National Human Genome Research Institute, 2021a,b; Understanding Genetics, 2021). Among those requiring in-person attendance, two addressed students and/or teachers (Yale University, 2016; National Human Genome Research Institute, 2021e) and one involved also the general population (Genome, 2021; Supplementary Table 3). As for the initiatives aimed at teachers and/or students, the National Human Genome Research Institute (NHGRI) in the United States offered to science teachers a short course in genomics during the summer of 2019 in order to improve their knowledge on the field (National Human Genome Research Institute, 2021e). Furthermore, Yale University (United States), during the second Pathways to Genomics and Proteomics Day in 2016, allowed 25 middle and high school students to spend a day focused on omics sciences and PM, with explanations and interactive activities about genomics (Yale University, 2016). Similarly, the exhibition “Genome: Unlocking Life’s Code” (Genome, 2021), held in 2013, also addressed the general population and was realized to celebrate the 10th anniversary of the completion of the Human Genome Project. In addition, lectures, symposia, and discussion groups were developed with the aim of exploring the topics of the exhibition and are available to watch on YouTube (Supplementary Table 4). Among the identified web-based resources, four addressed students and/or teachers (Genetic Science Learning Center of University of Utah Health Sciences, 2015, 2018; Department of Education of the American Museum of Natural History, 2021; GenomeQuébec, 2021), while 15 addressed the general population (HudsonAlpha, 2011, 2021; 23andMe, 2021; Cold Spring Harbor Laboratory, 2021a,b; Columbia University Medical Center Division of Molecular Genetics, 2021; DiseaseInfoSearch, 2021; Genes in Life, 2021; Genetic Literacy Project, 2021; iBiology, 2021; MyGenome App, 2021; National Center for Advancing Translational Sciences, 2021; National Human Genome Research Institute, 2021a,b; Understanding Genetics, 2021; Supplementary Table 3). As for the former, we identified the online course “Genetics, Genomics, Genethics” (Department of Education of the American Museum of Natural History, 2021), held in October 2019, that targeted middle and high school teachers and focused on the relationships between genetics and genomics and the legal, social, and ethical aspects. On the other hand, the Genetic Science Learning Center of University of Utah Health Sciences (United States) set up two websites – Teach.Genetics (Genetic Science Learning Center of University of Utah Health Sciences, 2015) and Learn.Genetics (Genetic Science Learning Center of University of Utah Health Sciences, 2018) – aimed at teachers and students, respectively. Both offer a vast choice of information and resources to support teaching in topics related to genomics and PM. Similarly, on the website of the non-profit organization GenomeQuébec (Canada), there is a platform for the education of high school students, mostly focused on basic genetic concepts (GenomeQuébec, 2021).

Among the 19 web-based resources aimed at the general population, the Educational Resources (National Human Genome Research Institute, 2021b), Fact Sheets about Genomics (National Human Genome Research Institute, 2021c), Talking Glossary of Genetic Terms (National Human Genome Research Institute, 2021f), and Introduction to Genomics (National Human Genome Research Institute, 2021d) sections on the website of the NHGRI (United States) are aimed at informing citizens about genetics and genomics (details in Supplementary Table 3). In addition, to celebrate the 15th anniversary of the completion of the Human Genome Project, the NHGRI launched in April 2018 a campaign called “15 for 15” (National Human Genome Research Institute, 2021a), explaining 15 ways genomics transformed and is transforming the world. We identified additional web resources aimed at improving citizens’ knowledge of genetics and genomics through readings or multimedia activities, including “DNA from the Beginning” (translated also in languages other than English) (Cold Spring Harbor Laboratory, 2021a), the application for mobile devices and the website of iCell (HudsonAlpha, 2021) ICell), the website of the private company 23andMe (2021), the platform iBiology (iBiology, 2021), the platform Genes in Life (2021), the website Learning Genetics (Columbia University Medical Center Division of Molecular Genetics, 2021), the interactive application GenomeCache (available for Apple devices) (HudsonAlpha, 2011), the application MyGenome App (available for Apple iPads) (MyGenome App, 2021), and the website Eugenics Image Archive (focused on the American eugenics movement) (Cold Spring Harbor Laboratory, 2021b; Supplementary Table 3).

Also, the website Understanding Genetics: Ask-a-Geneticist (Understanding Genetics, 2021) reports questions about genetics by individuals living all over the world, with related answers by graduate and postdoctoral fellows of the Department of Genetics of Stanford University (United States).

In addition, we identified two web resources focused on rare diseases. The first one is the Genetic and Rare Diseases Information Center (GARD, United States) (National Center for Advancing Translational Sciences, 2021), which aims to provide reliable, high-quality, simple, and updated information regarding rare diseases through its website, in English and Spanish. The second one is the website DiseaseInfoSearch (United States) (DiseaseInfoSearch, 2021), which contains a database of more than 10,000 diseases, including genetic ones.

Lastly, we identified the Genetic Literacy Project (GLP, United States) (Genetic Literacy Project, 2021), which is a non-profit association and includes also the Epigenetics Literacy Project and the Genetic Expert News Service (GENeS). The final aim of the association is to promote the diffusion of knowledge about human, animal, and plant genetics and genomics among the general population through the publication on its website of informative articles and videos addressing citizens’ literacy in these topics.

### Italian Initiatives

The search engine in Italian language produced a total of 13 initiatives carried out in Italy, whose details are reported in Supplementary Tables 5, 6 (European Researchers’ Night, 2021; Fondazione Telethon, 2021; Genetica biologia e salute, 2021; Istituto Italiano per la Medicina Genomica, 2021; Istituto Superiore di Sanità, 2021; Muse- Museo delle Scienze di Trento, 2021; Museo Tridentino di Scienze Naturali, 2021; Palazzo delle Esposizioni, 2021; Polo d’Innovazione di Genomica Genetica e Biologia, 2021; Portale Italiano delle Malattie Complesse, 2021; Scienze a Scuola, 2021; Università della Calabria, 2021; Zanichelli Aula di scienze, 2021).

Among them, eight required in-person attendance (European Researchers’ Night, 2021; Genetica biologia e salute, 2021; Istituto Italiano per la Medicina Genomica, 2021; Muse- Museo delle Scienze di Trento, 2021; Museo Tridentino di Scienze Naturali, 2021; Palazzo delle Esposizioni, 2021; Polo d’Innovazione di Genomica Genetica e Biologia, 2021; Università della Calabria, 2021), while five were web-based resources (Fondazione Telethon, 2021; Istituto Superiore di Sanità, 2021; Portale Italiano delle Malattie Complesse, 2021; Scienze a Scuola, 2021; Zanichelli Aula di scienze, 2021; Supplementary Table 5). Among the eight initiatives requiring physical attendance, four addressed students and/or teachers (Genetica biologia e salute, 2021; Istituto Italiano per la Medicina Genomica, 2021; Polo d’Innovazione di Genomica Genetica e Biologia, 2021; Università della Calabria, 2021), three addressed the general population (European Researchers’ Night, 2021; Muse- Museo delle Scienze di Trento, 2021; Palazzo delle Esposizioni, 2021), while one addressed both (Museo Tridentino di Scienze Naturali, 2021; Supplementary Table 4).

In Italy, a number of initiatives aimed at students and/or teachers took place in recent years (Supplementary Table 5). The most recent one is the project “High School Open Days Terni” (Polo d’Innovazione di Genomica Genetica e Biologia, 2021), which took place in Central Italy (Terni, Umbria Region) for 3 days in May 2019. The project aimed to make high school students learn about the research facility named “Polo d’Innovazione di Genomica, Genetica e Biologia” in the city of Terni. Other similar activities included “Vivere la scienza” (“To live science”) (Istituto Italiano per la Medicina Genomica, 2021) that took place in Turin (Piemonte Region) in 2018, consisting of interactive activities and laboratories that allowed students to carry out experiments focused on specific genetic topics, such as DNA fingerprinting, enzymes (e.g., β-galactosidase), DNA extraction, PCR technique, and genetic polymorphisms. Similar initiatives were the “Genetica, biologia e salute” (“Genetics, biology, and health”) (Genetica biologia e salute, 2021), held in Trento (Trentino Alto Adige Region) in 2009 and addressed middle and high school teachers, with the aim of improving their knowledge in the genetic field. Lastly, an initiative that took place in Southern Italy, titled OpenLab (Università della Calabria, 2021), was undertaken in 2017 by the University of Calabria. OpenLab is an interactive laboratory project funded by the Italian Ministry of University and Research and addresses middle and high school students, aiming to make students learn more about molecular genetics and the human genome.

Among the initiatives aimed at the general population, we identified two exhibitions organized by two Italian museums: the first, entitled “Genoma umano. Quello che ci rende unici” (“Human genome. What makes us unique”) (Muse- Museo delle Scienze di Trento, 2021), held in Trento in 2019 and the second one, called “DNA. Il grande libro della vita da Mendel alla genomica” (“DNA. The great book of life from Mendel to genomics”) (Palazzo delle Esposizioni, 2021), held in Rome in 2017.

The exhibition in Trento merged biological themes, such as DNA, genetic traits, mutations, and DTC-GTs, with a humanistic and artistic language. The aim of the exposition was to stimulate the public’s interest in such topics while paying attention to the ethical, social, and legal implications (ELSI) as well. On the other hand, the Roman exhibition was focused on the general aspects of genetics/genomics and was supplemented by a series of meetings and seminars open to the public.

Furthermore, among the events promoted by the 2019 and 2018 editions of the project “European Researchers’ Night” (European Researchers’ Night, 2021) in Italian cities, there were some focused on omics sciences. They are reported in detail in Supplementary Table 6.

Lastly, five web-based resources were identified (Fondazione Telethon, 2021; Istituto Superiore di Sanità, 2021; Portale Italiano delle Malattie Complesse, 2021; Scienze a Scuola, 2021; Zanichelli Aula di scienze, 2021; Supplementary Table 5). Among them, two addressed students and/or teachers, namely, the projects “Scienze a Scuola” (“Science at school”) and “Aula di Scienze” (“Science classroom”) (Scienze a Scuola, 2021; Zanichelli Aula di scienze, 2021), while three addressed the general population, which are the “Portale Italiano delle Malattie Complesse” (“Italian Portal of Complex Diseases”) (Portale Italiano delle Malattie Complesse, 2021), “Info_rare” (Fondazione Telethon, 2021), and “ISSalute” (Istituto Superiore di Sanità, 2021). These initiatives aim, through dedicated platforms, to provide citizens with useful information on rare and complex genetic diseases. Lastly, in 2009, an event titled “Bioweek: La nuova biologia per la salute della persona e del pianeta” (“Bioweek: the new biology for the health of the person and of the planet”) (Museo Tridentino di Scienze Naturali, 2021) that included a series of public events, such as seminars, round tables, public performances, and entertainment, was organized in Trento. This initiative addressed a wide public, including healthcare professionals, students, teachers, and the general population, with the aim of informing about the recent progress and developments of the health sciences and the impact of new biological knowledge on human health and the environment.

## Discussion

The need to inform and educate citizens in the omics sciences is a natural consequence of the disruptive development in this field since the sequencing of the human genome (International Human Genome Sequencing Consortium, 2004) imposes the necessity to identify the best tools, in terms of effectiveness and costs, to reach this goal. The aim of our study was to summarize the initiatives aimed at improving citizens’ literacy in omics sciences that can be retrieved over the web. Even though the aim was not to assess which countries are at the forefront in citizen engagement on omics sciences, the results suggest that greater attention to this topic is paid in the United States, although the results might be influenced by the search strategy adopted. Most companies providing new technologies such as DTC-GTs are based in the United States, which could be a possible explanation to the greater effort put in place for informing and educating citizens. This is further confirmed by a recent research showing that individuals educated in the United States had a significant better knowledge compared to those in other countries (Chapman et al., 2019). As for Italy, the relevance of increasing citizens’ literacy in omics sciences was recognized by authorities through specific national policies implemented over recent years, such as the National Guidelines on Public Health Genomics and the National Plan for Innovation of the Health System based on omics sciences, hence paving the way for specific research projects in this field (Presidenza del Consiglio dei Ministri, 2013; Boccia et al., 2014, 2017; Gazzetta Ufficiale, 2018).

From our study, we reported that a relevant number of initiatives addressed students and/or teachers, who have a crucial role in the spread of knowledge among the youth, revealing particular interest toward the education of future generations. In particular, almost half of the Italian initiatives and one-third of the non-European ones targeted the school population, underlining the importance of informing and educating young individuals. On the other side, all of the European initiatives included in our study were directed to the general population.

As for the type of identified initiatives, a few required in-person attendance, while most of them were web-based resources. In detail, more than half of the European initiatives and more than two-thirds of the non-European ones were web-based. As desirable, given the considerable development and growing use of the Internet and social networks in recent decades, this further underlines proper consideration of such information means, which could make it possible to reach especially younger groups of the population (Anderson and Jiang, 2018). On the contrary, Italy showed a different tendency, with more than half of the identified Italian initiatives requiring in-person attendance, suggesting the need to strengthen the use of digital means for public outreach, even if not neglecting the importance of events with in-person attendance. Both initiatives requiring physical attendance and web-based instruments identified through our search were highly heterogeneous. In detail, the former included exhibitions, seminars, courses, symposia, informative stands in public places, guided visits to research laboratories, and interactive laboratories, while the latter included web pages for reading and consultation by the public, online lectures and courses, web portals aimed at giving information and advice, videos and short movies, animations, and apps for mobile devices.

The heterogeneity among the retrieved resources is further confirmed by the topics addressed. Indeed, even though most of the initiatives focused on basic concepts of cellular biology, genetics, and genetic risks of diseases, some of them paid attention to more specific and complex topics as well, such as modern genome sequencing techniques, genetic tests, and the CRISPR method.

Such heterogeneity of both methods and dealt topics was found for all categories of initiatives included in our study, namely, European, non-European, and the Italian ones. This suggests that the landscape of topics dealt by citizen engagement initiatives in omics sciences, even if largely limited to genomics, is currently vast. In addition, several methods might be useful and effective to improve citizens’ literacy in this field; however, quantitative research is needed for a more accurate comparison.

Our work is the first attempt to summarize past and ongoing initiatives addressing citizens in the omics sciences field using a web search with a systematic and scientific approach. These results might be useful as a knowledge base for the design of future educational efforts. As for Italy, in particular, well-designed initiatives and strategies are requested to implement the National Plan for Innovation of the Health System based on omics sciences (Boccia et al., 2017).

### Study Limitations

Our study has several limitations. In particular, the use of only English and Italian languages for our search limited the chance to identify initiatives carried out in countries in which the first language is different. A broader search of initiatives or institutions addressing public engagement more generally in health or health-related research might have allowed us to identify further initiatives, web tools, activities, or events dealing with omics sciences as well. It should also be noted that many events might have not been advertised over the web, thus minimizing the chance for us to identify them through our search. In addition, even though the search engines employed in our study are the most used worldwide, the addition of other search engines or means of communications, such as Baidu or WeChat, might have led to the identification of further initiatives, especially in Eastern countries. To this end, our search strategies might have led to results skewed toward Western countries, with Eastern countries not being represented in our results. Thus, this could limit the comprehensiveness of our findings.

Furthermore, the great heterogeneity between the retrieved resources and sometimes the lack of relevant information did not allow us to perform a precise comparison, even though we reported qualitative information and possible similarities and differences. In addition, due to the lack of data on quantitative measures and indicators reported on the websites, we could not perform a comparison of the effectiveness of the retrieved initiatives on citizens’ literacy improvement.

## Conclusion

Awareness of existing citizen educational initiatives in the field of omics sciences performed so far is essential to design future ones. In our study, we summarized the characteristics of all the events available on the web that can be used as a knowledge base to implement further citizen educational campaigns and initiatives. Nowadays, increasing citizens’ literacy in omics science represents a priority for public health since more informed citizens are expected to make more appropriate choices about their health, thus having a positive impact on health systems. Further research is needed, however, in order to assess quantitatively the effectiveness of the different citizen engagement strategies in improving citizens’ literacy, for example, assessing the level of knowledge or awareness of omics sciences before and after the initiative using discussion groups, questionnaires, and similar methods.

## Data Availability Statement

The original contributions presented in the study are included in the article/Supplementary Material, further inquiries can be directed to the corresponding author.

## Author Contributions

SB and GC conceived the study. MS contributed to the study design. GC and MS identified the initiatives and extracted information from the websites/reports, critically discussed and interpreted the results of the review, and contributed equally to the drafting of the manuscript. SB critically reviewed the manuscript. All authors approved the final version.

## Conflict of Interest

The authors declare that the research was conducted in the absence of any commercial or financial relationships that could be construed as a potential conflict of interest.

## Footnotes

1 https://www.google.com/advanced_search
