# Supplementary material for: A Web Screening on Educational Initiatives to Increase Citizens’ Literacy on Genomics and Genetics
Source: Front Genet. 2021 Jul 7;12:637438. doi: 10.3389/fgene.2021.637438 (PMC8292827; doi:10.3389/fgene.2021.637438)
Supplement: Supplementary file 1 [file Data_Sheet_1.PDF]

SUPPLEMENTARY MATERIAL

Table 1. European educational initiatives (excluding Italy).

Table 2. Events held in Europe in the “European Researchers’ Night” – 2019 AND 2018 edition.

Table 3. Educational initiatives carried out in non-European countries.

Table 4. Events related to the “Genome: Unlocking Life's Code” exhibition.

Table 5. Educational initiatives carried out in Italy.

Table 6. Educational events held in Italy in the “European Researchers’ Night” – 2019 and 2018 edition.

Table 1. European educational initiatives (excluding Italy).

Name of the initiative [ref]	Country	Target population	Organizer/promoter	Topics	Type of initiative	Web page
European Researchers' Night - 2019 and 2018 editions [16] *	Europe, various Countries and cities	General population	European cities and/or institutions, funded by European Commission	Specified in table 2	Specified in table 2	https://ec.europa.eu/research/mariecurieactions/news/2018/european-researchers-night-2018-2019_en<br><br>https://ec.europa.eu/research/mariecurieactions/actions/european-researchers-night_en
¿Quieres visitar Navarrabiomed? [17] *	Spain	General Population	Navarrabiomed	Organization of a research laboratory, genetic research	Guided visit to the research centre	https://www.navarrabiomed.es/es<br><br>https://www.facebook.com/navarrabiomed/photos/a.319998134775248/2427132040728503/?type=3&theater
Your Genome [18] #	United Kingdom	General population	Public Engagement Team and Wellcome Genome Campus	Genetics and Genomics	Website with videos, animations, and interactive activities	www.yourgenome.org
GenoME [19] #	United Kingdom	General population	Personal Genome Project UK	Genetic code of four ambassadors of the Personal Genome Project UK and related traits; musical interpretation of genetic code	Application for Apple iPad	https://itunes.apple.com/gb/app/genome/id1358680703?mt=8<br><br>https://bmcmedgenomics.biomedcentral.com/articles/10.1186/s12920-018-0423-1
Orphanet [20] #	40 European and non-European Countries	General population	Institut national de la santé e de la recherche médicale (INSERM, France)	Rare diseases and related responsible genes, orphan drugs, patient associations, clinics and laboratories of excellence for specific rare diseases, on-going research projects, clinical trials, biobanks	Informative web pages	www.orpha.net

*: initiative requiring physical presence; #: web-based initiative

Table 2. Events held in Europe in the “European Researchers’ Night” – 2019 AND 2018 edition [16].

Project name	City (Country)	Year	Description of the events	Web page
Explorathon	Edinburgh (UK)	2019	Activities, talks, tours and lay posters detailing the genetics research undertaken within the MRC Institute of Genetics and Molecular Medicine in Edinburgh	https://www.explorathon.co.uk/wp-content/uploads/2019/09/Explorathon-2019-Flyer-EDINBURGH-WEB-9.9.19.pdf
LIFE LAB	Cambridge (UK)	2019	"Genomics! The Musical!": a series of musical mini-lectures to explore concepts of genetics and genomics	https://www.camlifelab.co.uk/cambridge
Probe	Dublin (Ireland)	2019	Public meetings about DNA and genetics, and about neurogenomics	https://www.tcd.ie/research/probe/programme/
QRIUS	Heidelberg (Germany)	2019	Public meetings about genetics and DNA and interactive laboratories where participants carried out easy experiments on DNA	https://www.nacht-der-forschung-heidelberg.de/events/?action=tribe_geosearch&tribe_paged=1&tribe_event_display=past&featured=false&is_recurrence_list=false
Science4Future	Kiel (Germany)	2019	Public meetings about genetics and the interaction of lifestyle factors with genes	https://www.wissenschafftzukunft-kiel.de/nacht-der-wissenschaft/programm-2019.html
Małopolska	Krakow (Poland)	2018	Demonstrations, laboratories, and conferences open to the public and focused on DNA, genes, cancer genetics, genetically modified organisms, transgenic plants	http://nocnaukowcow.malopolska.pl/
Explorathon	St. Andrews (UK)	2018	Public debate with experts about risks and benefits of sharing of genomic data, privacy and data use issues	https://twitter.com/ernscot?lang=en https://www.explorathon.co.uk/
LIFE LAB	Cambridge (UK)	2018	“Genomics & Us: The Future Of Genomic Medicine”: meeting focused on changes that genomics will determine on society and medicine in the next 10 years	https://www.camlifelab.co.uk/cambridge
Probe	Cork (Ireland)	2018	“DNAwesome!”: laboratory in which simple experiments (e.g. DNA extraction from fruit) were carried out.<br>Public meeting about neurogenetics and impact of the implementation of knowledge in this field on neurological development disorders (e.g. autism, learning difficulties, neurodevelopmental delay, psychosis)	https://www.tcd.ie/research/probe/programme/
MADRIDERN	Madrid (Spain)	2018	Seminar and following laboratory activity about CRISPR technique and ethical implications of its use.<br>Event on nutrigenetics, in which key topics in this field were presented with an audiovisual and interactive path; followed by laboratory activities with the participation of visitors	http://www.madrimasd.org/lanochedelosinvestigadores2018/

Table 3. Educational initiatives carried out in non-European countries.

Name of the initiative [ref]	Country	Target population	Organizer/promoter	Topics	Type of initiative	Web page
Short Course in Genomics [33] *	United States of America	Middle and high school teachers	National Human Genome Research Institute (NHGRI)	Genomics, complex diseases, genome sequencing, brain and behaviour, gene editing, bioinformatics, microbiome, ethical issues in genomic research	Short course lasting 4 days	https://www.genome.gov/careers-training/Professional-Development-Programs/NHGRI-Short-Course-in-Genomics
Pathways to Genomics and Proteomics Day – 2016 [21] *	United States of America	Middle and high school students in Yale Pathways to Science program	University of Yale	Omics sciences and personalised medicine	Lecture with an introduction on human genome, interactive activities and demonstrations. Visit to Yale Center for Genome Analysis.	https://westcampus.yale.edu/news/exploring-omics-west-campus-pathways-science-day?refresh=2<br><br>https://onhsa.yale.edu/news/exploring-omics-west-campus-pathways-science-day
Genome: Unlocking Life's Code [22] *	United States of America	General population, students, and teachers	Collaboration between Smithsonian's National Museum of Natural History (NMNH) and National Human Genome Research Institute (NHGRI)	Genetics, genomics, and history of discoveries about DNA	Exhibition, multimedia contents on its website, meetings, symposia, lectures, discussion groups	https://unlockinglifescode.org/<br><br>https://www.genome.gov/outreach/unlocking-lifes-code-exhibition
Genetics, Genomics, Genethics [36] #	United States of America	Primary, middle, and high school teachers	Department of Education dell'American Museum of Natural History	Relationships between genetics, genomics, and ethical, social, and legal implications	Online course with lessons, essays, case studies, book readings, video, interactive activities, picture galleries	https://www.amnh.org/learn-teach/seminars-on-science/courses/genetics-genomics-genethics
Teach.Genetics [39] #	United States of America	Teachers	Genetic Science Learning Center of University of Utah Health Sciences	Genetics, biology, and health; including epigenetics, gene therapy, personalised medicine	Tools and activities to aim teachers in their classroom activities	https://teach.genetics.utah.edu/

Learn.Genetics [40] #	United States of America	Students	Genetic Science Learning Center of University of Utah Health Sciences	Genetics, biology, and health; including epigenetics, gene therapy, personalised medicine	Articles, pictures, animations, interactive activities, and videos to aim study	https://learn.genetics.utah.edu/
Website of GenomeQuébec [41] #	Canada	High school students	GenomeQuébec	Genetics, genomics	Articles, pictures, animations	http://www.genomequebec-education-formations.com/education-en
Website of the National Human Genome Research Institute of the National Institutes of Health [37,55–57] #	United States of America	General population	National Human Genome Research Institute (NHGRI)	Genomics, diseases	Fact sheets, genetic glossary, articles, pictures, and animations	https://www.genome.gov/About-Genomics/Educational-Resources<br><br>https://www.genome.gov/about-genomics/fact-sheets<br><br>https://www.genome.gov/genetics-glossary<br><br>https://www.genome.gov/About-Genomics/Introduction-to-Genomics
15 for 15 [38] #	United States of America	General population	National Human Genome Research Institute (NHGRI)	15 ways genomics transformed and is transforming the world (e.g. genome sequencing, genetic variations, human origins, agriculture, DTC-GTs and others)	Informative web pages	https://www.genome.gov/dna-day/15-for-15
DNA From The Beginning [42] #	United States of America	General population	Funded by The Josiah Macy, Jr. Foundation; created by DNA Learning Center, Cold Spring Harbor Laboratory	Genetics (three areas: classic genetics, molecular genetics, control and organization of genes)	Animations, picture galleries, video-interviews, biographies	www.dnaftb.org
iCell [26] #	United States of America	General population	HudsonAlpha	Biology, with hints about DNA	Interactive simulation allowing the exploration of an animal, vegetal, or bacterial cell	http://icell.hudsonalpha.org/

Website of the private company 23andMe [27] #	United States of America	General population	23andMe	Genes, polymorphisms, origins, phenotype	Articles	https://www.23andme.com/en-int/gen101/
iBiology [28] #	United States of America	General population	National Science Foundation, National Institute of General Medical Sciences, Lasker Foundation	Biology, including genomics (e.g. microRNA, telomerase, CRISPR technique)	Videos	https://www.ibiology.org
Learning Genetics [32] #	United States of America	General population	Columbia University Medical Center, Division of Molecular Genetics	Genetics, genetic tests, exome sequencing	Videos	http://learninggenetics.org/index.html
Genes in Life [29] #	United States of America	General population	Genetic Alliance	Impact and influence of genes on life, family, disease occurrence; reasons and usefulness of genetic counselling; genomic research	Articles, videos	http://GenesInLife.org
GenomeCache [34] #	United States of America	General population	HudsonAlpha	Genomics	Application with articles and reading, fun facts, questions with related score	https://itunes.apple.com/us/app/genomecache/id470275184?mt=8
MyGenome App [23] #	United States of America	General population	Illumina	Effect of genetic variants on health and response to drugs, and mechanisms of their transmission to offspring	Application with articles and readings, and animations	https://emea.illumina.com/clinical/clinical_informatics/mygenome_app.html?langsel=/it/
Eugenics Image Archive [24] #	United States of America	General population	Cold Spring Harbor Laboratory	History of American eugenics movement	More than 2,500 pictures with textual descriptions	http://www.eugenicsarchive.org/eugenics/

Understanding Genetics: Ask-a-Geneticist [25] #	United States of America	General population	Stanford University	Genetics	Questions about genetics by individuals all over the world, with related replies by graduate and post-doc fellows of the Department of Genetics	http://genetics.thetech.org/ask-a-geneticist
Website of the Genetic and Rare Diseases Information Center (GARD) [31] #	United States of America	General population	National Center for Advancing Translational Sciences (NCATS), funded by NCATS and National Human Genome Research Institute (NHGRI)	Rare and genetic diseases	For every disease, there are a description, information about diagnosis and treatment, clinical trials, patient organizations, scientific articles and papers.<br>In English and Spanish.	https://rarediseases.info.nih.gov/
DiseaseInfoSearch [30] #	United States of America	General population	Genetic Alliance, creato tramite finanziamenti federali	Diseases, including genetic ones	Database about more than 10,000 diseases. For each of them there is a description, information on support groups, clinical trials, scientific articles, and other resources	http://diseaseinfosearch.org/
Website of the Genetic Literacy Project (GLP) [35] #	United States of America	General population	Genetic Literacy Project	Human, animal, and plant biology	Informative articles and videos	www.geneticliteracyproject.org

*: initiative requiring physical presence; #: web-based initiative

Table 4. Events related to the “Genome: Unlocking Life's Code” exhibition.

Genome: Unlocking Life's Code Exhibition Closing Symposium: Genomics and Global Health: What does the future hold?	https://www.genome.gov/27558981/genome-unlocking-lifes-code-exhibition-closing-symposium
A Spectrum of Perspectives: Native Peoples and Genetic Research	https://www.youtube.com/playlist?list=PLS6nSmuURFJC6iY2lWMFwfVjXDHfC6AwC
Q?rius Presentation: What Exactly is the Human Genome?	https://www.youtube.com/watch?v=R1cOqafAbhk
Finding our Inner Neanderthal: Evolutionary Geneticist Svante Pääbo's DNA Quest	https://www.youtube.com/watch?v=zL_d7b1bsUE
Q?rius Presentation: The Dog Genome: Shedding Light on Human Disease	https://www.youtube.com/watch?v=sxtX1pfSUec
Q?rius Presentation: Know Your Family History - Improve Your Health	https://www.youtube.com/watch?v=LigpJTh3ahw
Is Genetic Information Different?	https://www.youtube.com/playlist?list=PL1ay9ko4A8smgQqLL46-ouK8Pk1HrNoJB

Table 5. Educational initiatives carried out in Italy.

Name of the initiative	City (Region)	Target population	Organizer/promoter	Topics	Type of initiative	Web page
High School Open Days Terni [47] *	Terni (Umbria)	High school students	Polo d'Innovazione di Genomica, Genetica e Biologia; City Hall of Terni	Malaria and gene editing technologies for its treatment	Guided visits, seminars, videos	http://www.comune.terni.it/news/facciamo-conoscere-il-polo-di-genomica-agli-studenti# https://www.pologgb.com/high-school-open-days-terni/
Vivere la scienza [43] *	Turin (Piemonte)	Students aged 17-19 years	Istituto Italiano per la Medicina Genomica (IIGM)	DNA Fingerprinting, experiments about enzymes (e.g. beta galattosidase), DNA extraction, PCR technique, genetic polymorphisms	Interactive laboratories	https://www.iigm.it/site/index.php?id=144&t=articolo https://www.iigm.it/oggetti/273_VIVERE%20LA%20SCIENZA.pdf https://www.iigm.it/oggetti/274_Vivere%20la%20scienza%20Volantino.pdf
Openlab [50] *	Rende (Calabria)	Middle and high school students	University of Calabria	Genomics, molecular genetics	Interactive laboratory with practical experiments	http://www.openlab.unical.it/
Genetica, biologia e salute [51] *	Trento (Trentino Alto Adige)	Middle and high school teachers of scientific area	Museo Tridentino di Scienze Naturali, in collaboration with the University of the Studies of Trento and the Centro Interdipartimentale per la Biologia Integrata (CIBIO)	Cellular genetics, physiology, embryonic development, genetic engineering, basic concepts of gene therapy, and strategies for the development of new drugs	Education and update course	http://www.mtsn.tn.it/perlascuola/archivio-aggiornamento.asp http://www.mtsn.tn.it/perlascuola/documenti/2009/prog_corso_agg_genetica_lug09.pdf
Bioweek: "La nuova biologia per la salute della persona e del pianeta" [52] *	Trento (Trentino Alto Adige)	Specialists, students, teachers, and citizens	Museo Tridentino di Scienze Naturali, in collaboration with the University of the Studies of Trento and the Fondazioni di Ricerca Trentine	Progress in the fields of biology and genomics, and their impact on health and environment	Public events involving seminars, round tables, art, entertainment	http://www.molecularlab.it/eventi/evento.asp?n=440
Genoma umano. Quello che ci rende unici [48] *	Trento (Trentino Alto Adige)	General population	Museo delle Scienze (MUSE)	DNA, genetic traits and mutations, DTC-GTs	Exhibition	https://www.muse.it/it/Esplora/mostre-temporanee/Archivio/Pagine/Genoma-umano.aspx

DNA. Il grande libro della vita da Mendel alla genomica [49] *	Rome (Lazio)	General population	Palazzo delle Esposizioni in Rome	Genetics and genomics	Exhibition and meetings/seminars for deeply discuss topics of the exhibition	https://www.palazzoesposizioni.it/mostra/dna-il-grande-libro-della-vita-da-mendel-alla-genomica
European Researchers' Night – 2019 and 2018 editions [16] *	Italy, various cities	General population	Italian cities and/or institutions, funded by European Commission	Specified in table 5	Specified in table 5	https://ec.europa.eu/research/mariecurieactions/actions/european-researchers-night_en https://ec.europa.eu/research/mariecurieactions/news/2018/european-researchers-night-2018-2019_en
Scienze a Scuola [53] #		Middle and high school students and teachers	Scientific high school "G. Galilei" in Ancona (Marche)	In the section of biology there are lessons about the history of discoveries about DNA, about genetic code, RNA translation, genetics and its history	Lessons with related tests, scientific glossary	www.scienzeascuola.it
Aula di Scienze [54] #		Teachers and students	Zanichelli editore S.p.A.	Latest news and discoveries in the field of science, including biology and genomics	Web pages for reading/consultation	https://aulascienze.scuola.zanichelli.it/
Portale Italiano delle Malattie Complesse [44] #		General population	Section of Hygiene of the Institute of Public Health of Università Cattolica del Sacro Cuore, Rome – project funded by National Centre for Disease Prevention and Control (CCM) of the Italian Ministry of Health	Multifactorial and complex diseases, and laboratories offering genetic testing in Italy	Web pages for reading/consultation	www.pimaco.it
Info_rare [45] #		General population	Telethon Foundation	Rare genetic diseases, on-going clinical studies, and rare disease patient associations	E-mail consultation with specialist physicians in genetics, who reply to information requests by patients or citizens, even about on-going research projects	http://www.telethon.it/cosa-facciamo/per-i-pazienti/info-rare
ISSalute [46] #		General population	Istituto Superiore di Sanità (ISS)	Causes, symptoms, treatment, and prevention of diseases, including few due to genetic alterations	Encyclopaedia with more than 1,700 pages	www.issalute.it

*: initiative requiring physical presence; #: web-based initiative

Table 6. Educational events held in Italy in the "European Researchers' Night" – 2019 and 2018 edition [16].

Project name	City (Region)	Year	Description of the events	Web page
ERN-Apulia	Bari (Apulia)	2019	Interactive laboratories and explanations about DNA and genetics	https://www.laricercaviendinotte.it/bari/
ERN-Apulia	Lecce (Apulia)	2019	Interactive laboratories about DNA and genetics for children aged 7 years or older	https://www.laricercaviendinotte.it/lecce/
ERN-Apulia	Foggia (Apulia)	2019	Public meeting about genes, cancer, and environmental exposures	https://www.laricercaviendinotte.it/wp-content/uploads/2019/10/programma-unifg-ita-23.09.2019-_low.pdf
Meet	Milan (Lombardy)	2019	Public meeting about new discoveries in the field of personalized medicine and the importance of citizen and patient engagement	http://www.meetmetonight.it/la-settimana-di-meetmetonight/
Sharper	Cagliari (Sardinia)	2019	Public meeting about DNA, genes, and genetics	http://www.sharper-night.it/wp-content/uploads/2019/09/Sharper_CAGLIARI-programma-2019-OK.pdf
Sharper	Catania (Sicily)	2019	Playful activities aimed at improving participants' knowledge about rare genetic diseases;<br>public dialogue on hereditary cancer syndromes;<br>interactive activities on the relation between genes and diet	http://www.sharper-night.it/wp-content/uploads/2019/09/Sharper_CATANIA-programma-2019-OK.pdf
Sharper	Palermo (Sicily)	2019	Public meeting about genetics and cystic fibrosis;<br>dialogue about the use of genetics in forensic medicine	http://www.sharper-night.it/wp-content/uploads/2019/09/Sharper_PALERMO-programma-2019-OK.pdf
Sharper	Pavia (Lombardy)	2019	Visit to a genetic research centre with explanations about discoveries in molecular genetics and their impact in the development of new treatments for rare disease, neurodegenerative disorders, and cancer	http://www.sharper-night.it/wp-content/uploads/2019/09/Sharper_PAVIA-programma-2019-OK.pdf
Sharper	Perugia (Umbria)	2019	Event about DNA and genetic laboratory techniques	http://www.sharper-night.it/wp-content/uploads/2019/09/Sharper_PERUGIA-programma-2019-OK.pdf
Society	Bologna (Emilia-Romagna)	2019	Public dialogue about DNA and genetics	http://nottedeiricercatori-society.eu/notte-2019/
Bright	Siena (Tuscany)	2018	Public meetings on genetics and biotechnologies	http://www.bright-toscana.it/wp-content/uploads/2018/09/Magazine_2018.pdf
ERN-Apulia	Bari (Apulia)	2018	Public meeting on DNA, microbiome and other topics	https://www.laricercaviendinotte.it/ern2018/bari/

B-Future	Caserta (Campania)	2018	Meeting with researchers about epigenetics, role of Mediterranean diet in disease prevention, digital imaging, e-health applications	http://nottedeiricercatori.neuromed.it/
Bees	Rome (Lazio)	2018	– Exhibition focused on rare diseases, disability, DNA addressing children aged 6-11 years;<br>– Interactive laboratory open to the public and focused on DNA;<br>Visit to research centres open to the public and focused on the interaction of environmental factors (e.g. lifestyles) and DNA	https://www.frascatiscienza.it/pagine/notte-europea-dei-ricercatori-2018/programma/?evs=2090&bytarget=&bycategory=&bytown=&byday=&byorgs
Meet	Milan (Lombardy)	2018	Events (informative stands) grouped under "personalized medicine" label	http://www.meetmetonight.it/wp-content/uploads/2018/09/programma-milano-2018_agg.pdf
Sharper	Trieste (Friuli Venezia Giulia)	2018	Events focused on experiments on DNA and cancer	http://www.sharper-night.it/wp-content/uploads/2018/09/SHARPER_Trieste-2018-def.pdf
